# The Formyl Peptide Receptor 2 as a Target for Promotion of Resolution of Inflammation

**DOI:** 10.2174/1570159X20666220913155248

**Published:** 2023-05-18

**Authors:** Ewa Trojan, Monika Leśkiewicz, Enza Lacivita, Marcello Leopoldo, Agnieszka Basta-Kaim

**Affiliations:** 1 Laboratory of Immunoendocrinology Department of Experimental Neuroendocrinology, Maj Institute of Pharmacology, Polish Academy of Sciences, Kraków, Poland;; 2 Department of Pharmacy - Drug Sciences, University of Bari, via Orabona 4, 70125 Bari, Italy

**Keywords:** Neuroinflammation, resolution of inflammation, formyl peptide receptors, pro-resolving agonists, SPMs (small pro-resolving mediators), lipoxin

## 1 INTRODUCTION

Recently, our thinking about inflammation and its resolution (resolution of inflammation, RoI) and mechanisms controlling the course of this process has changed [1]. Since the phases of inflammation do not develop sequentially but overlap, the interest in the formyl peptide receptor-2 (FPR2) is strongly justified because it appears to regulate immune response and its resolution. Accordingly, it may represent a molecular target for innovative pharmacological approaches to inflammation-based disorders. FPR2 and the other subtypes: FPR1 and FPR3, belong to the G-protein coupled receptor family [2]. The expression of FPRs was demonstrated on several immune cells, including neutrophils and monocytes/macrophages. In the brain, FPR2 is expressed in astrocytes, neurons, and microglia [3]. Thereby FPR2 plays an important role in the various central nervous system (CNS) diseases, including Alzheimer's disease, Parkinson’s Disease, Multiple Sclerosis, and peripheral chronic inflammatory illnesses [4-6]. Moreover, FPR’s role in cardiovascular pathologies and cancer progression is strongly postulated [7].

Interestingly, FPR2 is a highly promiscuous receptor. It can interact with chemically diverse endogenous or exogenous ligands, including peptides (*e.g*., serum amyloid A) [8, 9] and eicosanoids (*e.g*., lipoxin A4 and aspirin-triggered lipoxin, (AT-LXA4)) [10] as well as synthetic molecules (*e.g*., substituted quinazolinone Quin-C1, MR-39) [11-16]. FPR2 activation can stimulate signal transduction pathways, depending on the ligand's concentration and the cell type involved. It has been found that FPR2 is an unconventional receptor not only due to the diversity of its ligands but also because its activation translates into opposite biological effects. Generally, it shows an immunomodulatory role by regulating pro-resolving and anti-inflammatory, but also pro-inflammatory activities. Importantly, NADH oxidase (NOX; nicotinamide adenine dinucleotide oxidase) seems to be one of the crucial regulators of inflammatory pathways [17]. In fact, FPR2 can modulate oxidative stress through NOX-dependent reactive oxygen species (ROS) generation, whose dysregulation in inflammatory diseases has been demonstrated [7]. Furthermore, it has been found that in some conditions, FPRs could be involved in ROS generation through the interaction with the urokinase (uPA)/urokinase receptor (uPAR) systems and, consequently, activation of the Rac1 (Ras-related C3 botulinum toxin substrate 1) and ERK (Extracellular Signal-regulated Kinase) intracellular pathways [18]. This versatile role renders FPR2 an attractive and challenging therapeutic target.

## 2 PEPTIDE AND LIPIDS FPR2 AGONISTS

N‐formyl‐methionyl‐leucyl‐phenylalanine (fMLF) – a chemoattractant tripeptide derived from *E. coli* is a full FPR2 agonist [19]. Apart from fMLF, synthetic hexapeptides like WKYMV bind to FPR2 with high efficiency and activate neutrophil and monocyte functions, including chemotaxis, mobilization of complement receptor-3, cytokine release as well as activation of NADPH oxidase [20]. Annexin A1 (AnxA1) and its mimetic N-terminal peptide Ac2-26 are also FPR2 agonists in promoting RoI. In fact, after binding to FPR2, AnxA1 modulates microglial phagocytosis and limits the duration of the inflammatory response [21]. Moreover, AnxA1 strongly activates FPR2 by transducing specific downstream signals through NOX-dependent ROS generation [17].

While considering the pathophysiological role of FPR2, the diverse group of amyloidogenic proteins associated with chronic inflammation and/or amyloidosis should be stressed. Among them, serum amyloid A (SAA), β-amyloid peptide 42 (Aβ42), and a peptide fragment of the aberrant human prion protein (PrP106-126) are the most studied. The mechanisms of their action are not fully understood. Nevertheless, these peptides stimulate FPR2 on monocytes, triggering chemotaxis and release of TNF-α and IL-1β [22].

Furthermore, FPR2 was reported to interact with a lipid metabolite – lipoxin A4 (LXA4)- the first described endogenous ligand of this receptor. Interestingly, its isolation in 1984 by Serhan *et al.* [23] resulted in the discovery of this receptor and started the development of new pharmacological agents. LXA4 is a member of the family of lipoxins generated from arachidonic acid (AA) *via* 5-, 12-, and 15 lipoxygenase (LOX). It is an unusual metabolite of AA with anti-inflammatory and immunoregulatory biological functions. Both LXA4 and its analog AT-LXA4 mediate RoI by breaking the signals in inflammation and promoting phagocytosis of nonphlogistic apoptotic neutrophils, consequently leading to the silencing of the inflammation [10, 24]. Furthermore, LXA4 decreases reactive oxygen species (ROS) and pro-inflammatory cytokines and chemokines production. The anti-inflammatory response mediated by LXA4 engages several intracellular signaling pathways, such as the transcription factors: AP-1 (Activator protein 1), NF-кB (nuclear factor kappa light chain enhancer of activated B cells), or Nrf (nuclear erythroid 2-related factor 2) [25]. Additionally, LXA4 activates peroxisome proliferator-activated receptor (PPAR) gamma, which is another important player in controlling inflammation [26]. Among others, it has been shown that LXA4 inhibits microglia activation after hemisection of the spinal cord [27]. In rats with hemorrhage, the beneficial anti-inflammatory effects of LXA4 were related to its influence on the MAPK signaling pathway [28]. Moreover, Wu *et al.* [29] showed the anti-inflammatory effect of AT-LXA4 in astrocyte and microglia cell cultures stimulated with bacterial endotoxin (lipopolysaccharide, LPS). Unfortunately, LXA4 and AT-LXA4 are characterized by poor bioavailability. Both agonists are rapidly inactivated *in vivo* by the metabolic enzyme system, which somewhat limits the possibility of their wider application. Moreover, there is no direct evidence that LXA4 can cross the blood-brain barrier [30, 31].

Recently a lot of data underlined that resolvins are the crucial class of FPR2 agonists engaged in the pro-resolving and anti-inflammatory regulation of immune response. Among them, series D resolvins (RvD1) derived from docosahexaenoic acid (DHA) are of special interest because they are recognized as the missing link explaining the health-promoting effects of DHA in the diet [32]. Generally, the role of RvD1 is to inhibit the migration of inflammatory cells, stimulate macrophages to phagocytosis of apoptotic neutrophils, and inhibit NF-кB activation and secretion of pro-inflammatory cytokines [33]. Li *et al.* demonstrated the anti-inflammatory and pro-resolving effects of RvD1 in PC12 cell cultures as increased expression of anti-inflammatory microglia markers. Moreover, alternative microglia activation was correlated with the activation of STAT6 and the PPAR-γ signaling pathway [34]. The authors observed the potential therapeutic efficacy of RvD1 *in vitro* and *in vivo* models of Parkinson's disease. This positive effect was connected with the inhibition of the TLR4/NF-кB pathway [35].

## 3 NON-PEPTIDE ALX/FPR2 AGONISTS

As stated above, lipoxins and resolvins exert strong endogenous anti-inflammatory effects. Nevertheless, their unfavorable pharmacokinetic properties represent a limitation of further studies, mostly *in vivo* experiments and clinical trials [36]. Therefore, great emphasis is placed on discovering new agonists less susceptible to metabolic deactivation with a longer biological half-life [35].

**Fig. (1):**
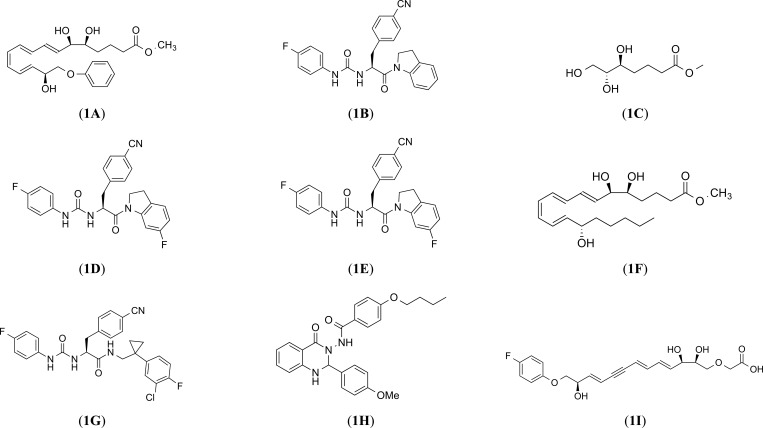
Structires of selected examples of the FPR2 ligands.

Compound **1** (*e.g*. 16-phenoxy-LXA4) (Fig. **1A**) [13] is well accepted to represent the 1^st^ generation of lipoxin analogs. However, its therapeutic potential was limited due to rapid *in vivo* clearance after oral or intravenous administration. In turn, the 2^nd^ generation of lipoxin analogs, Compound **2** (*e.g*., ZK-994) (Fig. **1I**), showed a better therapeutic potential in a few models of inflammation [37], is generation led to the development of the 3^rd^ generation analogs (*e.g*., Compound **43**) (Fig. **1E**). This compound was characterized by anti-inflammatory properties and was able to attenuate LPS-induced NF-κB activation with a potency similar to LXA4 [38, 39]. Notwithstanding, pharmaceutical companies and academia have constantly been working on discovering new FPR2 agonists with promising therapeutic potential. These studies described several small-molecule FPR2 agonists, *e.g*., BML-111 (Fig. **1C**), which can reduce inflammation and simultaneously potentiate the release of anti-inflammatory factors (*e.g*., IL-4, IL-10) in numerous inflammatory-based diseases [35, 40].

Ye *et al.*, in the rat model of focal cerebral ischemia-reperfusion, demonstrated that LXA4 analog – LXA (4) (Fig. **1F**) ME inhibited microglia activation and the expression of pro-inflammatory mediators (TNF-α, IL-1β) while increasing the anti-inflammatory cytokines (IL-10, TGF-β) release [41]. Finally, the observations of the research group by He *et al.* led to the demonstration that Quin-C1, (Fig. **1H**) a quinazoline derivative is a highly selective FPR2 agonist characterized by anti-inflammatory properties [42].

It should be noted that our group also contributed to the field of FPR2 agonist studies by discovering a series of ureidopropanamide-biased agonists, exemplified by MR39 (compound (*S*)-17 in [13]), which shows favorable pharmacokinetic and anti-inflammatory properties. Accordingly, MR39 (Fig. **1G**) lowered IL-1β and TNF-α levels in LPS-stimulated primary microglial cells [13]. Moreover, MR39 exerted neuroprotective effects in LPS-stimulated rat primary microglial cells at doses higher than LXA4 but lasting longer. Additionally, this pro-resolving effect was mediated by the inhibition of ERK1/2 and NF-κB pathways [43]. What is more, the observed effects were FPR2-mediated because they were not observed in organotypic hippocampal cultures (OHCs) obtained from FPR2 knock-out (FPR2^-/-^) mice and/or were abolished by pre-treatment with the FPR2 antagonist - WRW4 in OHCs from wild-type mice [15]. MR39 revealed similar anti-inflammatory, and neuroprotective effects in OHCs stimulated β-amyloid, being able to reduce the release of pro-inflammatory mediators (IL-1β, IL-6, TNF-α) induced by β-amyloid and improve the release of anti-inflammatory mediators (IL-4, TGF-β) [14]. Nevertheless, the concentration of MR39 needed to demonstrate this beneficial pro-resolving and anti-inflammatory effect is relatively high; therefore, a question arose about the risk of side effects *in vivo* studies. Given these limitations, our recent research has led to the subsequent optimization of MR39 structure and the identification of compounds AMS21 (Fig. **1B**) and CMC23 (Fig. **1D**) (compounds (*S*)-11e and (*S*)-11l in [16]). These compounds show neuroprotective properties in an experimental model of inflammation, reducing the levels of pro-inflammatory cytokines (TNF-α, IL-1β) in LPS-stimulated primary microglial cell culture. At the same time, these compounds showed good pharmacokinetic properties (metabolic stability, ability to cross the blood-brain barrier) and inhibited caspase-3 activity induced by LPS in the concentration ranges as the endogenous SPMs [16]. Hence, it will be beneficial to address this topic in an animal model *in vivo* to investigate the complex and physiological role of FPR2 modulation, which is of key significance in neuroinflammation and RoI.

## CONCLUSION

The discovery that RoI is a highly coordinated and active process controlled by endogenous pro-resolving mediators has highlighted FPR2 as an important potential molecular target able to affect the intensity of inflammation and alleviate chronic inflammatory diseases. To date, several studies in different animal models of neuroinflammation have shown that administration of LXA4, an endogenous agonist of FPR2, could be useful to inhibit microglial activation and attenuation of neuroinflammation, thus opening the door to new therapeutic strategies for the treatment of CNS disorders. For this reason, many researchers aim to find FPR2 ligands having optimized pharmacodynamic and pharmacokinetic properties suitable for *in vivo* studies. So far, several FPR2 agonists have been reported in the literature characterized by a wide range of FPR2 potency, metabolic stability, and anti-inflammatory and pro-resolving properties. However, since no unequivocal effectiveness of any of them has been demonstrated and the research has not brought us much closer to identifying such a promising find, further consistent searches seem to be of key importance in developing new therapeutic options to treat brain disorders characterized by persistent neuroinflammation.

## LIST OF ABBREVIATIONS

AA: Arachidonic Acid
CNS: Central Nervous System
FPR2: Formyl Peptide Receptor-2
OHCs: Organotypic Hippocampal Cultures
PPAR: Peroxisome Proliferator-Activated Receptor
ROS: Reactive Oxygen Species
SAA: Serum Amyloid A
uPAR: Urokinase Receptor
